# Targeted inhibition of methicillin-resistant *Staphylococcus aureus* biofilm formation by a graphene oxide-loaded aptamer/berberine bifunctional complex

**DOI:** 10.1080/10717544.2022.2079768

**Published:** 2022-05-26

**Authors:** Yi Ning, Xiaoqi Wang, Pingan Chen, Shiwu Liu, Jue Hu, Rong Xiao, Ling Li, Fangguo Lu

**Affiliations:** a Department of Microbiology, The Medicine School of Hunan University of Chinese Medicine, Changsha, Hunan, People’s Republic of China; b Experimental Center of Molecular Biology, The Chinese Medicine School of Hunan University of Chinese Medicine, Changsha, Hunan, People’s Republic of China

**Keywords:** Drug-resistance bacteria, MRSA, anti-biofilm, targeted therapy, graphene oxide-loaded aptamer/berberine bifunctional complex

## Abstract

Biofilm formation is known to promote drug resistance in methicillin-resistant *Staphylococcus aureus* (MRSA), which is closely related to persistent infections in hospital settings. In this study, a DNA aptamer specific to penicillin-binding protein 2a (PBP2a) with a dissociation constant (*K*_d_) of 82.97 ± 8.86 nM was obtained after 14 cycles of systematic evolution of ligands by exponential enrichment (SELEX). Next, a bifunctional complex containing the aptamer intercalated by berberine into the double-strand region was prepared and adsorbed onto the surface of graphene oxide **(**GO) by π-stacking interactions. The GO-loaded aptamer/berberine bifunctional complex showed significantly higher inhibition of MRSA biofilm formation than the control. Furthermore, this study shows that the complex possesses anti-biofilm activity, which can be attributed to the ability of the aptamer to reduce cell-surface attachment by blocking the function of PBP2a and berberine to attenuate the level of the accessory gene regulator (*agr*) system, which plays an important role in mediating MRSA biofilm formation. Therefore, the simultaneous delivery of berberine and PBP2a-targted aptamer using GO may have potential for the treatment of chronic infections caused by MRSA biofilms. It also provides a new avenue for multitarget treatment of bacterial biofilms.

## 1. Introduction

With the abuse of antibacterials, various drug-resistant bacteria, including methicillin-resistant *Staphylococcus aureus* (MRSA), have emerged (Hassoun et al., 2017; Lakhundi & Zhang, 2018). MRSA, an important nosocomial and community-acquired pathogen, is responsible for serious infections resistant to conventional antibiotic therapy, such as pneumonia, infective endocarditis, soft tissue infections, bacteremia, and osteomyelitis (Lee et al., 2018; Ju et al., 2021). In addition to its resistance to β-lactam antibiotics, MRSA forms a biofilm that endows it with multidrug resistance (Hosseini et al., 2020; Cascioferro et al., 2021). Bacterial biofilms are microbial communities that are attached to a solid surface using extracellular polymeric substances, which display an important barrier to antibiotic treatment and host defense. The tolerance of bacterial biofilms to antibiotics is 1000-fold higher than that of planktonic bacteria. To date, the bacterial factors associated with biofilm formation, accumulation, and regulation among clinical MRSA isolates remain largely unknown. Initial attachment is necessary for biofilm formation. It has been reported that polysaccharide of intercellular adhesion (PIA), encoded by *icaADBC* locus, is well-known factor associated with biofilm formation (Formosa-Dague et al., 2016). In addition, it has been demonstrated in recent years that PBP2a is also related to the formation of biofilms by mediating the cell-surface attachment (Santiago et al., 2015). Biofilm formation is regulated by a two-component quorum-sensing (QS) system encoded by the *agr* locus. The autoinducing peptide (AIP) is a signaling molecule in the *agr* system. When cell density increases, AIP production is upregulated, thereby inducing biofilm formation (Tan et al., 2018). Therefore, the development of anti-biofilm agents that block the function of PBP2a and interfere with the *agr* system would be a sensible strategy for the development of new treatments for recalcitrant MRSA infections.

Aptamers, also called chemical antibodies, are single-stranded DNA (ssDNA) or RNA sequences obtained through the systematic evolution of ligands by exponential enrichment (SELEX) (Kaur, 2018; Lyu et al., 2021). They can fold into secondary and three-dimensional structures, allowing them to recognize a wide range of target molecules with high affinity and specificity, such as proteins, small molecules, metal ions, bacterial cells, viruses, and cancer cells (Takahashi et al., 2015; Liu & Zhao, 2017; Bayat et al., 2018; Sun et al., 2019; Jamal et al., 2020; Yang et al., 2021). Despite the relative youth of the aptamer field, the distinctive features of aptamers make them attractive tools for use in a wide array of applications, ranging from diagnostics to therapeutics (Bala et al., 2018; Xie et al., 2021). The use of aptamers as therapeutic reagents and inhibitors has gradually become a popular research field. Pegaptanib, a 27-nt RNA aptamer that specifically binds to vascular endothelial growth factor (VEGF), is the first therapeutic aptamer approved by the US Food and Drug Administration as an antiangiogenic medicine that can treat neovascular age-related macular degeneration by inhibiting intraocular blood vessel growth (Moshfeghi & Puliafito, 2005). AS1411, an unmodified guanosine-rich 26-mer DNA oligonucleotide that specifically binds to nucleolin, is the first aptamer to enter clinical trials for cancer treatment via mechanisms affecting cancer cell growth, proliferation, and survival (Mongelard & Bouvet, 2010). The use of aptamers as therapeutic antagonists for infectious disease treatment is a rapidly growing field of research. Our group screened aptamer 3, which can recognize the functional domains of flagellin of *Salmonella choleraesuis*, leading to the suppression of biofilm formation by blocking tropism and the initial attachment mediated by flagella (Ning et al., 2015). Li et al. selected aptamer 1, which is specific to the hemagglutinin (HA) protein of the H1N1 influenza virus and can inhibit virus-induced hemagglutination and proliferation in host cells (Li et al., 2016). Additionally, several other aptamers have been developed as therapeutics for the treatment of *Mycobacterium tuberculosis*, HIV, human papilloma virus, and SARS-CoV-2 via mechanisms impairing the function of target proteins (Nicol et al., 2013; Srivastava et al., 2021; Sun et al., 2021; Ratanabunyong et al., 2022). Hence, the use of aptamers as inhibitors is a promising development with potentially extensive therapeutic benefits.

At present, increasing attention has been focused on traditional Chinese herbal medicines because of their low cost, good compatibility with the human body, and low side effects. Berberine, an isoquinoline-type alkaloid extracted from *Coptis chinensis* (Huanglian in Chinese), has been widely used to purge intense heat and remove toxicosis, and it has anticancer, anti-arrhythmia, antioxidant, and anti-inflammatory effects (Wang et al., 2017; Song et al., 2020; Poopedi et al., 2021; Rauf et al., 2021). Recently, berberine has also been used as a novel agent against a number of pathogenic microorganisms and their biofilms, such as *S. aureus*, *Salmonella typhimurium*, and *Mycobacterium abscessus* (Tseng et al., 2020; Bhatia et al., 2021; Xu et al., 2021). However, few studies have focused on the mechanisms of biofilm formation prevention, especially in the QS system. In addition, berberine has disadvantages of poor solubility and membrane permeability. Therefore, there is an urgent need to develop a new strategy for berberine delivery to enhance its intracellular accumulation and bioavailability and to further study the mechanisms underlying the effects of berberine on biofilm formation.

Graphene oxide (GO), a single atomic layered carbon material, has been used widely to construct carrier system for drug delivery owing to its advantages of low cytotoxicity, good biocompatibility, and stable storage (Sharma & Mondal, 2020; Grant et al., 2021). GO has an ultrahigh specific surface area for binding plenty of molecules, including nucleic acids, proteins, liposomes, and certain drugs via π-stacking interactions or chemical coupling (Kozik et al., 2019; Mousavi et al., 2019; Chaudhary et al., 2021). Importantly, GO can protect the molecules that bind to its surface from degradation or enzymolysis in a biological environment (Ning et al., 2017). Accordingly, a GO-based drug delivery system for transmitting aptamer/berberine bifunctional complex was developed to dispose of MRSA biofilms. The GO-loaded aptamer/berberine bifunctional complex displayed a higher suppression of biofilm formation against MRSA than the control. Moreover, a novel application of this complex was described, in which MRSA biofilm formation was suppressed by reducing cell-surface attachment and simultaneously restraining the expression of the *agr* system. This study not only realizes the loading of nucleic acid drug and traditional Chinese medicine monomer in the same drug delivery system but also provides an effective way for the multitarget treatment of biofilms.

## 2. Materials and methods

### 2.1. Materials and reagents

GO nanosheets were purchased from Xianfeng Materials Technology (Nanjing, China). All the DNA oligonucleotides used in this study were synthesized and purified by Shanghai Sangon Biotechnology Co., Ltd. (Shanghai, China). The transpeptidase region (extracellular region) of PBP2a was provided by Shanghai Sangon Biotechnology Co., Ltd. via the *Escherichia coli* prokaryotic expression system. SYBR Green qRT-PCR Kit and SYTO9 dye were obtained from Baiaolaibo Biotechnology Co., Ltd. (Beijing, China). UNIQ-10 Column TRIzol Total RNA Isolation Kit, ELISA plate, 96-well tissue culture plate, lambda exonuclease, and berberine were purchased from Shanghai Sangon Biotechnology Co., Ltd. All chemicals used were of analytical grade. All reagents were dissolved in ultrapure water. The MRSA strain was provided by the Medical Laboratory Center of the First Affiliated Hospital of Hunan University of Chinese Medicine (Changsha, China). *S. aureus*, *S. epidermidis*, *S. typhimurium*, and *E. coli* strains were obtained from Xiangya Hospital of Central South University (Changsha, China).

### 2.2. Apparatus

Ultrapure water was prepared using a Milli-Q water purification system (electrical resistivity of 18 MΩ cm^−1^). The fluorescence intensity was measured at 25 °C using an LS55 luminescence spectrometer (PerkinElmer, Wokingham, UK) in a quartz cell. The excitation and emission slits were both set with a bandpass of 10.0 nm. A U-1500 UV-vis spectrophotometer (Macy, China) was used to determine the absorbance of biofilms stained by crystal violet (CV). Confocal laser scanning microscopy (CLSM) (Nikon, Japan) and atomic force microscopy (AFM) (Bruker, USA) were used to analyze the distribution and thickness of the biofilm formed on the coverslips.

### 2.3. Selex procedure

A synthetic ssDNA pool containing 35-base central random sequences were flanked by primer sites on both sides: 5′-GGGAGCTCAGAATAAACGCTCAA-N_35_-TTCGACATGAGGCCCGGATC-3′ The forward and reverse primers used for amplification of the DNA pool were 5′-GGGAGCTCAGAATAAACGCTCAA-3′ and 5′-P-GATCCGGGCCTCATGTCGAA-3′, respectively. The procedure for screening DNA aptamers of PBP2a was based on our previously published work (Ning et al., 2014). The only difference was that the sub-library for the next selection round was obtained through lambda exonuclease digestion in the present study. The amount of ssDNA in each round was assayed using UV-Vis spectroscopy. The aptamer-binding ratio was determined by determining the fluorescence of the eluted ssDNA every two rounds. After 14 rounds of selection, the PCR products selected from the 14th round were subjected to high-throughput sequencing. The selected parameters for PBP2a are listed in Table S1.

### 2.4. Determination of dissociation constant and binding parameters of aptamer

Dissociation constants were obtained by calculating the number of aptamers bound to PBP2a from a calibration plot of the mean fluorescence intensity. The equation Y = BmaxX/(K_d_+X) was used to determine the dissociation constant (*K*_d_), where Bmax represents the maximal fluorescent intensity measured in this experiment, Y is the mean fluorescent intensity, and X is the concentration of the aptamer. For the sensitivity assay, various amounts of MRSA cells ranging from 800 to 10^7^ colony-forming units (CFU)/mL were incubated with 200 nM FAM-labeled aptamer 1. For specificity assay, MRSA and other bacteria (*S. aureus*, *S. epidermidis*, *S. typhimurium*, and *E. coli*) at 10^7^ CFU/mL were incubated with 200 nM FAM-labeled aptamer 1. After binding and washing, the bacterium-aptamer 1 complex sediment was resuspended in 100 μL of phosphate buffer saline. The binding affinity of aptamer 1 for various bacteria was determined by recording the fluorescence intensity at 514 nm with excitation at 480 nm.

### 2.5. Construction of the GO-loaded aptamer/berberine bifunctional complex

Various concentrations of aptamer 1 ranging from 0 to 280 nM were heated at 100 °C for 5 min and then immediately placed in iced water for 10 min. Next, 50 μg/mL of berberine was added to the treated aptamer 1 at different concentrations and incubated at 37 °C for 30 min to form the aptamer/berberine bifunctional complex. GO nanosheets were dissolved in ultrapure water and subjected to ultrasonication in an ice bath using an ultrasonic cell disruptor at 200 W, wherein the pulse was applied for 3 s and then switched off for 3 s. After 3 h, a homogeneous solution of GO was obtained. Different concentrations of GO (0–80 μg/mL) were injected into the formed aptamer 1/berberine bifunctional complex (200 nM aptamer 1) and incubated at 37 °C for 30 min. The fluorescence intensity was measured at 530 nm with excitation at 365 nm. The GO-aptamer 1/berberine complex was washed by centrifugal spin filtration and resuspended in Tris-HCl buffer (20 mM Tris-HCl, 5 mM MgCl_2_, pH 8.0) for use in subsequent experiments.

### 2.6. Effect of the GO-aptamer 1/berberine complex on MRSA biofilms formation

First, a CV assay was conducted to quantify biofilm formation. MRSA (10^7^ CFU/mL) was inoculated into a 96-well plate loaded with 200 μL nutrient medium per well, and then GO (60 μg/mL), aptamer 1(200 nM), berberine (50 μg/mL), aptamer 1 (200 nM)/berberine (50 μg/mL), and GO (60 μg/mL)-aptamer 1(200 nM)/berberine (50 μg/mL) were added individually. After culture at 37 °C for 48 h under static conditions, planktonic bacteria were discarded, and the plate was washed three times with normal saline. Next, 200 μL of 1% CV was injected into each well and incubated for 5 min at 25 °C. After decolorization with 200 μL 30% acetic acid for 10 min, the absorbance was measured at 595 nm. Second, the distribution of biofilms formed on the coverslips was analyzed by CLSM (488 nm, 600×). MRSA (10^7^ CFU/mL) was inoculated into 6-well plates containing sterile coverslips and loaded with 2 mL of nutrient medium. The same experimental parameters mentioned above were individually added and cultured at 37 °C for 48 h under static conditions. The coverslips were washed with normal saline three times, fixed with methanol for 30 min, and stained with SYTO9 in the dark for 15 min at 25 °C. After washing with normal saline three times, the coverslips were observed by CLSM. Finally, AFM was used to measure the thickness of the biofilm formed on coverslips after 48 h, as described above. The control received no treatment.

### 2.7. Measurement of biofilm after treatment with aptamer 1 and qRT-PCR analysis of agr gene transcription levels after treatment with berberine at different concentrations

MRSA cells (10^7^ CFU/mL) were inoculated in a 96-well plate loaded with 200 μL nutrient medium per well, and aptamer 1 was added at concentrations of 0–1400 nM. Subsequent procedures were performed according to the CV assay. Berberine at concentrations of 0–100 μg/mL was added to the wells. After culture at 37 °C for 48 h, planktonic cells were collected by centrifugation at 17,000 × *g* for 10 min at 4 °C. RNA extracted from the precipitated cells was used for qRT-PCR analysis. qRT-PCR was carried out by technicians of the public laboratory of Xiangya Hospital of Central South University in conformity with the procedure provided by the manufacturer; the data were also provided by them. The primers used for qRT-PCR analysis are listed in Table S2.

### 2.8. Statistical analysis

Statistical analyses were performed using SPSS version 25.0. One-way analysis of variance with *post-hoc* analysis using Dunnett’s *t*-test was performed to analyze the differences between groups, and the least significant difference test and Kruskal–Wallis *H* test were used to detect pairwise differences. Statistical significance was set at *p* < 0.05. All experiments were performed in triplicates and repeated three times.

## 3. Results and discussion

### 3.1. Aptamer selection

After 14 rounds of selection, the aptamers displayed strong affinity for the target protein. The enrichment of PBP2a-specific aptamers was assayed during the selection process, and increasing binding ratios of aptamers to PBP2a were observed after round 12–14 (Figure S1). The binding ratio of the DNA pool was much higher at round 12 than at round 10, whereas the percentage of bound DNA pool did not obviously increase after round 12. This result revealed that the affinity of the aptamers for PBP2a remained constant after round 12. This is attributed to the stringent pressure increase in the screening process, which continuously enriched the specific aptamers against PBP2a. Considering that the affinity at round 14 was slightly higher than that at round 12, the PCR products from the 14th round were sent for high-throughput sequencing. Two representative sequences were obtained, and the nominated aptamers were 1 and 2 (Table 1). It should be noted that the number of repetitions of these two aptamers appeared more than three times. For example, the aptamer 1 nucleotide sequence appeared 299 times, and aptamer 2 appeared 64 times. However, the number of repetitions of the other sequences was less than 3. This suggested that these two aptamers were continuously enriched by round selection.

**Figure 1.**
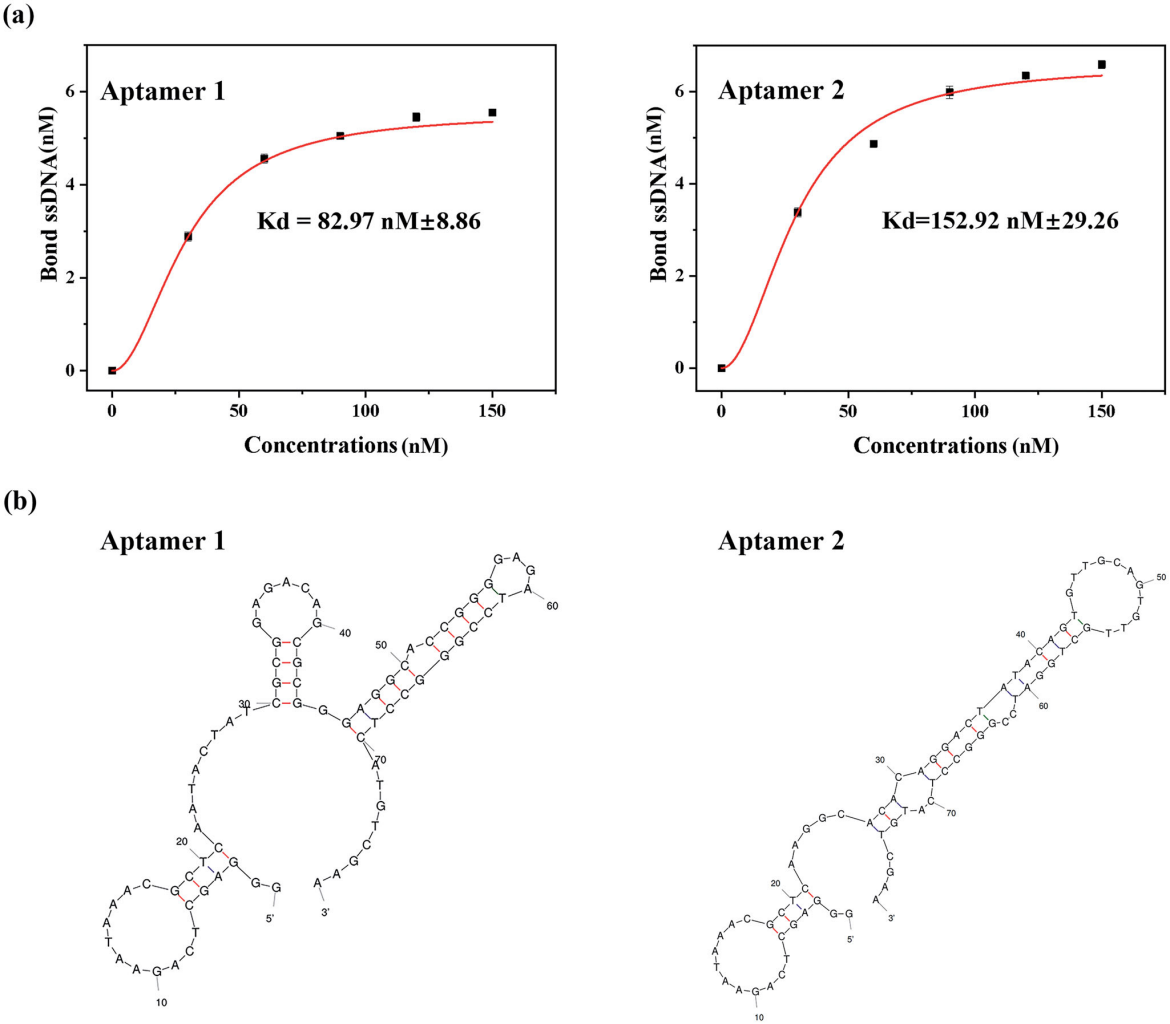
Determination of the dissociation constant and secondary structure prediction. (a) Fluorescence analysis to determine the dissociation constant (Kd) of selected aptamers1, 2. Aptamer 1 = 82.97 ± 8.86 nM, Aptamer 2 = 152.92 ± 29.26 nM; (b) The secondary structures of the two aptamers. The stem-loop structures might be potential binding sites. Error bars represent the standard deviations from three replicate measurements.

**Table 1.** Two representative aptamers from ssDNA library were selected via the SELEX.

Aptamer	Sequence (from 5′ to 3′)	Repeated times	*K*_d_ (nM)
aptamer1	GGGAGCTCAGAATAAACGCTCAATACTATCGCGGAGACAG	299	82.97 ± 8.86
	CGCGGGAGGCACCGGGGAGATCCGGGCCTCATGTCGAA		
aptamer2	GGGAGCTCAGAATAAACGCTCAAGGCACACAGGACTATAC	64	152.92 ± 29.26
	AGTGTTGCAGTGTTGCTGGATCCGGGCCTCATGTCGAA		

### 3.2. Dissociation constant assay and the binding parameters analysis

The binding affinities of the aptamers were analyzed by a fluorescent assay, and their dissociation constants peaked in the nanomolar range (Figure 1(a)). The Kd of aptamer 1 was 82.97 ± 8.86 nM, whereas that of aptamer 2 was slightly higher at 152.92 ± 29.26 nM. These results indicated that the aptamers had good potential to identify MRSA by effectively binding to PBP2a. The secondary structures of the two aptamers were predicted using the Mfold server software, and their typical stem and loop structures are displayed in Figure 1(b). Aptamers can bind to their targets by forming special secondary structures, such as stems, loops, hairpins, pseudoknots, and G-quadruplexes. The stem-loop structure of the two aptamers probably represents active sites for binding different epitopes of PBP2a. Additionally, it is assumed that the stem structures can maintain the stability of the secondary structure of aptamers, whereas the bases in the loops may be responsible for binding the target. In this study, aptamer 1, which showed the highest affinity, was selected for the next experiment. As shown in Figure S2, the fluorescence intensity increased with an increasing number of MRSA cells, ranging from 800 to 10^7^ CFU/mL. The linear range was 10^3–^10^7^ CFU/mL (*R*^2^=0.9945), and the detection limit reached 800 CFU/mL. In addition, the fluorescence intensity of MRSA was much stronger than that of the non-targeted bacteria (Figure S3) despite having equal numbers of cells. These findings suggested that aptamer 1 showed high sensitivity and selectivity toward MRSA, making it a good candidate for specific targeting of MRSA.

**Figure 2.**
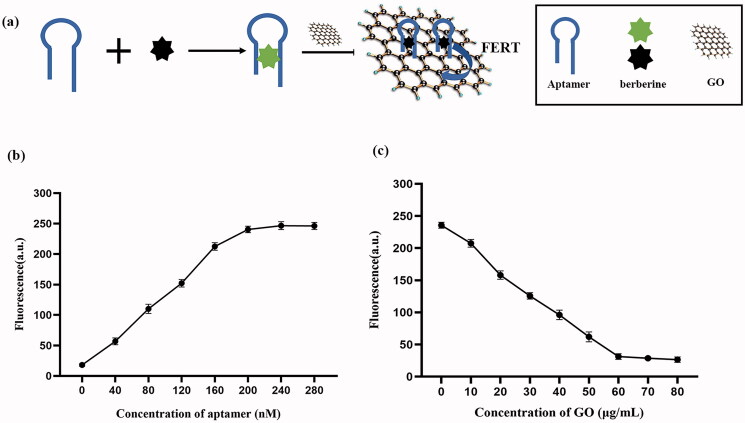
Fabrication of the GO-loaded aptamer 1/berberine bifunctional complex. (a) Schematic drepresentation of the bifunctional complex design; (b) Optimization of the ratio of berberine to aptamer 1; (c) Optimization of the concentration of GO. Error bars represent the standard deviations from three replicate measurements.

**Figure 3.**
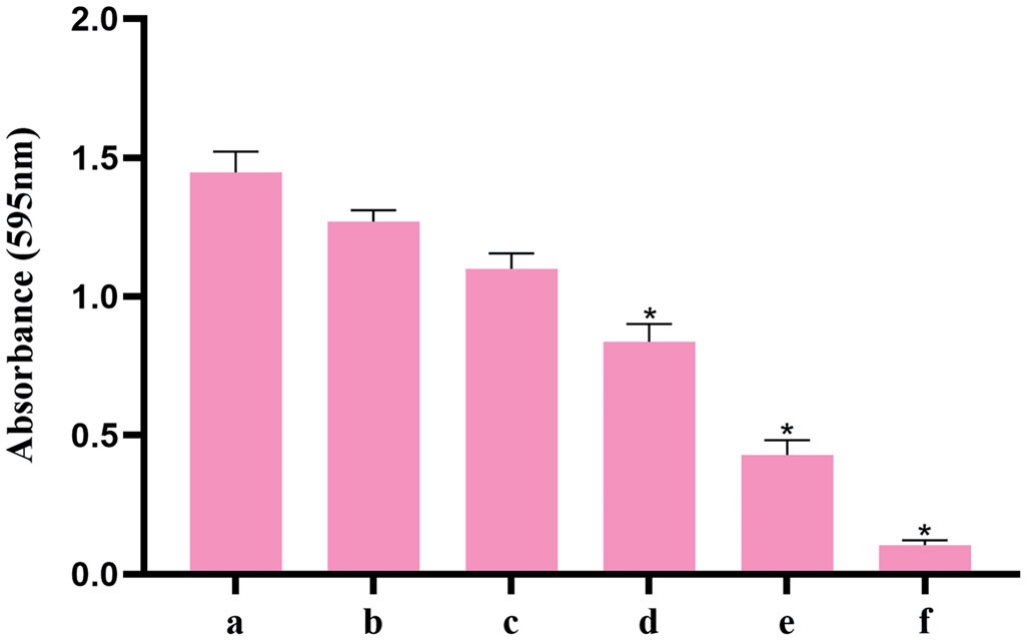
Crystal violet-stained biofilms of MRSA were determined by measuring the OD595 after adding 30% acetic acid. (a) control; (b) GO; (c) Aptamer 1; (d) Berberine; (e) Aptamer 1/berberine; (f) GO-Aptamer 1/berberine. Error bars represent the standard deviations from three replicate measurements. *Indicates *p* < 0.05 compared with the respective control group.

### 3.3. Fabrication of the GO-loaded aptamer 1/berberine bifunctional complex

To develop an efficient method for suppressing biofilm formation, a GO-loaded aptamer 1/berberine bifunctional complex was generated (Figure 2(a)). Similar to ethidium bromide, berberine showed low fluorescence when it was free. However, fluorescence increased significantly after the addition of aptamer 1. This indicated that berberine was inserted into the stem structure of aptamer 1, which led to the enhancement of fluorescence intensity. The fluorescence decreased when GO was added to the system, indicating that the aptamer 1/berberine complex was adsorbed onto GO via π-stacking interactions, and the fluorescence was quenched. Therefore, the ‘off-on’ and ‘on–off’ modes can be used to characterize the construction of a simultaneous delivery system. In addition, the optimal quality of aptamer 1 and GO was surveyed in this study. As shown in Figure 2(b), the fluorescence increased with increasing aptamer 1 concentration and stopped increasing when the aptamer 1 concentration reached 200 nM, indicating that 50 μg/mL berberine was bound to 200 nM aptamer 1. Figure 2(c) shows that GO concentration increased with decreasing berberine/aptamer 1 fluorescence intensity. Approximately 91.3% of fluorescence was quenched by 60 μg/mL GO, suggesting that 60 μg/mL GO could effectively quench the fluorescence of berberine/aptamer 1 (with 200 nM aptamer 1) via fluorescence energy resonance transfer (FERT). Therefore, a simultaneous delivery system based on GO for the targeted inhibition of MRSA biofilm formation was successfully designed using a self-assembly technology.

### 3.3. CV assay and microscopic observations

**Figure 4.**
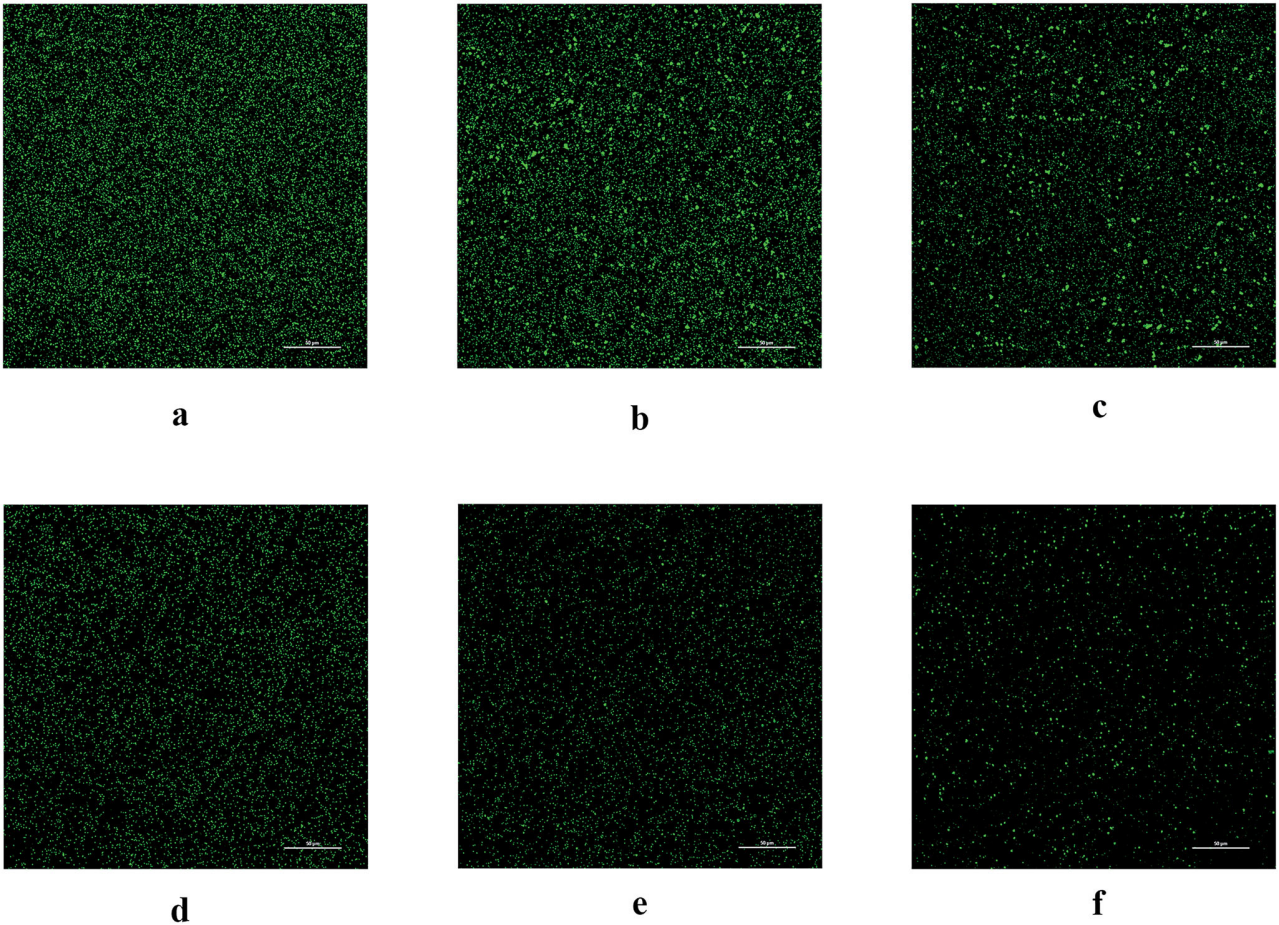
CLSM analysis of the distribution of MRSA biofilms under various conditions. (a) control; (b) GO; (c) Aptamer 1; (d) Berberine; (e) Aptamer 1/berberine; (f) GO-Aptamer 1/berberine. scale bar = 50 μm (CLSM, 600 times×).

**Figure 5.**
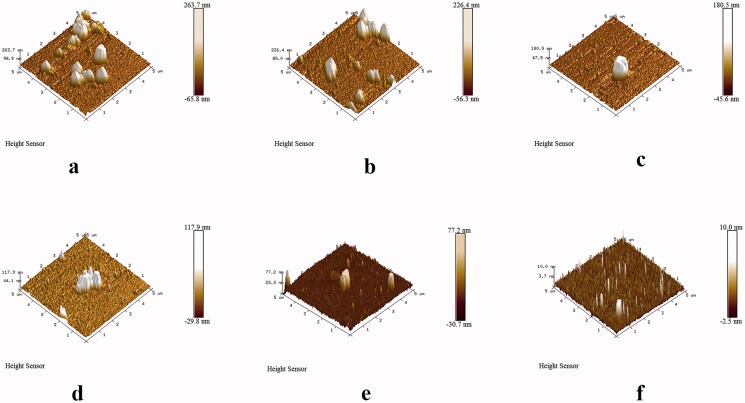
AFM analysis of the thickness of MRSA biofilms under various conditions. (a) control; (b) GO; (c) Aptamer 1; (d) Berberine; (e) Aptamer 1/berberine; (f) GO-Aptamer 1/berberine. Scale bar = 1.0 μm.

A CV assay was used to evaluate the inhibition of MRSA biofilm formation by this system (Figure 3). Compared with that in the control, approximately 92.4% of MRSA biofilm formed on the plates treated with 60 μg/mL GO, indicating that GO (60 μg/mL) showed low toxicity toward MRSA. However, 200 nM aptamer 1 or 50 μg/mL berberine alone was able to inhibit MRSA biofilm formation to varying degrees. The inhibition rates reached 20.8 and 41.2%, respectively. Additionally, the inhibition rate declined to 70.3% when the berberine/aptamer 1 (containing 200 nM aptamer 1) complex was added. These results indicated that aptamer 1 can improve the targeting rate of berberine and has a synergistic inhibitory effect with berberine. Moreover, the inhibition rate peaked at 92.8% after treatment with the GO-berberine/aptamer 1 (containing 200 nM aptamer 1) complex, suggesting that GO can improve the stability and availability of the berberine/aptamer 1 in complex in biological environments. These results were confirmed by microscopic observations. As shown in Figure 4, the biofilms in the control group were distributed evenly over the attachment surface, and maintained a complete and uniform biofilm structure. The biofilms treated with GO were almost identical to those in the control group. However, the multilayered biofilms covering the entire surface of the coverslips began to dissociate from their three-dimensional (3D) structures and presented a discrete distribution when berberine or aptamer 1 was used. A further decrease in the number of colonies was observed in the culture treated with the berberine/aptamer 1 complex, in which almost all MRSA cells existed in the form of planktonic bacteria. In the culture treated with the GO-berberine/aptamer 1 complex, few bacteria appeared on the surface of the coverslips, and the cells were relatively dispersed. Figure 5 shows the thicknesses of the biofilms formed on the coverslips, as measured by AFM. Compared with that in the control group and other experimental groups, the mean thickness of the biofilm treated with the GO-berberine/aptamer 1 complex greatly decreased, with a mean thickness of 12.8 ± 0.6 nm. The mean thicknesses of the biofilm at different conditions are shown in Table S3. These results showed that the biofilm-forming ability of MRSA was considerably impaired by the GO-berberine/aptamer 1 complex.

### 3.4. Application of aptamer 1 and berberine at different concentrations against different targets in MRSA biofilm formation

**Figure 6.**
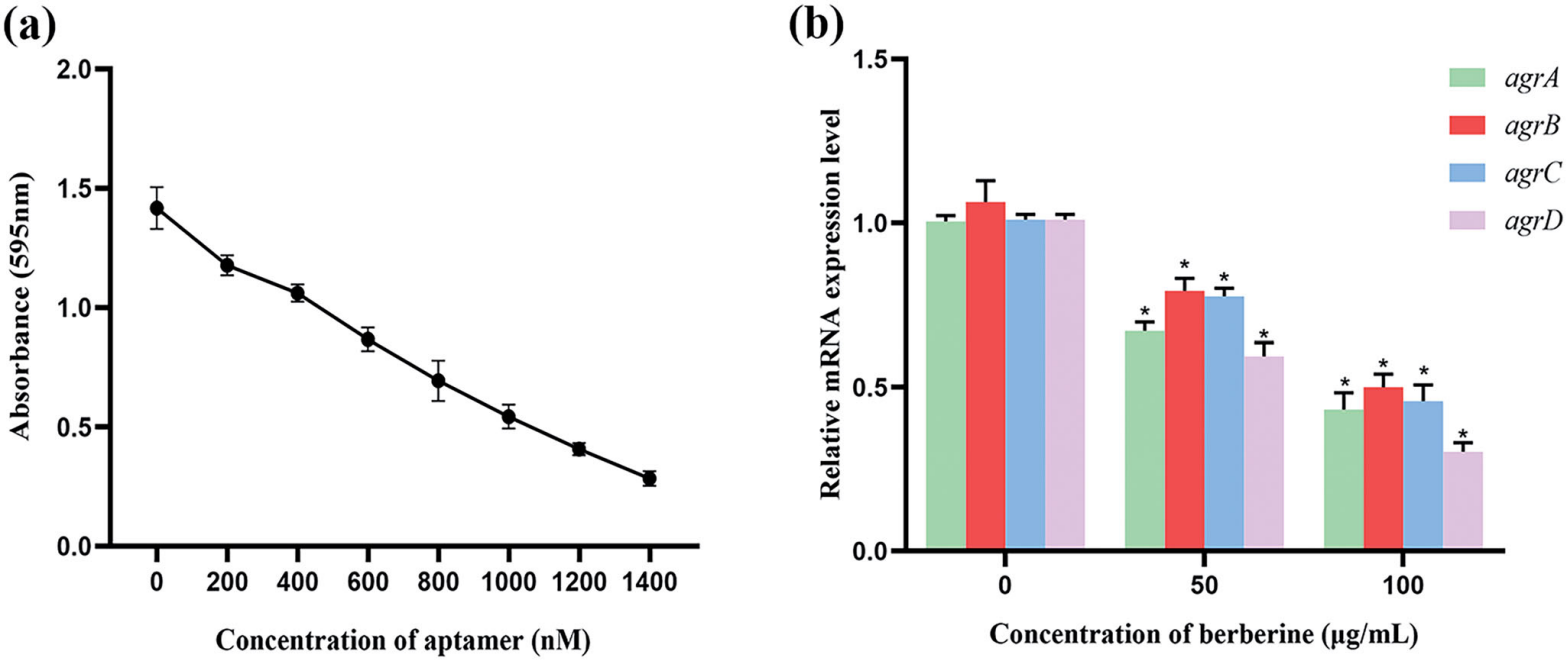
Application of aptamer 1 and berberine at different concentrations against different targets in MRSA biofilm formation. (a) Aptamer 1 ranging from 0 to 1400 nM were assayed by the crystal violet method; (b) Determinant of the four *agr* genes expression after treatment with berberine ranging from 0 to 100 μg/mL. Error bars represent the standard deviations from three replicate measurements. *Indicates *p* < 0.05 compared with the respective control group.

**Figure 7.**
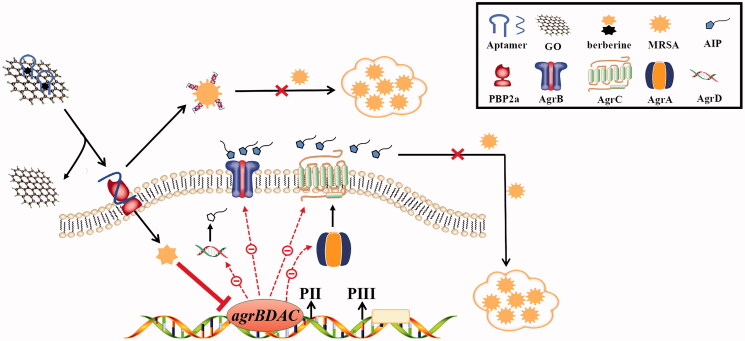
A model that the GO-loaded aptamer 1/berberine bifunctional complex suppressed the MRSA biofilm formation on an abiotic surface. PBP2a-mediated cell surface attachment and *agr* genes-triggered bacterial aggregation effect caused the MRSA to form mature biofilms in absence of this bifunctional complex. Upon adding the bifunctional complex, the MRSA biofilm formation was inhibited by simultaneously impairing PBP2a and *agr* genes.

PBP2a plays an important role in the initial stage of MRSA biofilm formation by mediating cell surface attachment. Therefore, PBP2a is a potential target for the inhibition of MRSA biofilm formation. As shown in Figure 6(a), the absorbance of CV at 595 nm gradually decreased as the concentration of aptamer 1 increased from 0 to 1400 nM. The higher the concentration of aptamer 1, the lower the MRSA biofilm formation. This revealed that aptamer 1 could bind specifically to PBP2a of MRSA, blocking its function and thus reducing biofilm formation. Figure 6(b) shows the relative expression levels of the four *agr* genes after treatment with berberine. Berberine at concentrations of 50 and 100 μg/mL inhibited the expression of four key genes in MRSA biofilm formation. When the berberine concentration was 100 μg/mL, the relative expression levels of these genes decreased to less than 50%. This indicated that berberine inhibited MRSA biofilm formation by downregulating the expression of the *agr* genes. Accordingly, the GO-berberine/aptamer 1 complex was assumed to effectively inhibit MRSA biofilm formation by simultaneously impairing PBP2a and *agr* genes. As depicted in Figure 7, when the GO-berberine/aptamer 1 complex acts on MRSA, early attachment is restricted by aptamer 1 via specific binding to PBP2a, covering its binding sites that attach to the matrix surfaces, whereas the downregulation of the expression of *agr* genes by berberine destroys the MRSA QS system by inhibiting AIP production. This makes it difficult for MRSA to produce a mature biofilm and causes most of the MRSA cells to remain in the planktonic state. Therefore, this bifunctional complex presents great potential for development as an effective inhibitor of MRSA biofilm formation.

## 4. Conclusions

We successfully developed a biofilm-targeted delivery platform based on GO coated with a berberine/aptamer 1 complex that can efficiently inhibit MRSA biofilm formation. In this drug delivery system, aptamer 1 serves two roles as a nucleic acid drug and a targeted delivery system for berberine. This is the first study investigating the simultaneous loading of nucleic acid drugs and traditional Chinese medicine monomers that target different key factors in MRSA biofilm formation in the same drug delivery system, which greatly enhanced the biofilm removal rate. Biofilms are closely related to multidrug resistance and persistent infection; therefore, there is an urgent need to develop effective methods for combating biofilm formation. Therefore, this novel strategy for suppressing biofilm formation is a promising approach for future research and development.
